# Correction to: Inhibition of PAD4 enhances radiosensitivity and inhibits aggressive phenotypes of nasopharyngeal carcinoma cells

**DOI:** 10.1186/s11658-021-00303-7

**Published:** 2021-12-30

**Authors:** Hao Chen, Min Luo, Xiangping Wang, Ting Liang, Chaoyuan Huang, Changjie Huang, Lining Wei

**Affiliations:** 1 Department of Oncology, The Second Nanning People’s Hospital, No. 13 Dancun Road, Jiangnan District, Nanning, 530031 Guangxi China; 2 Department of Endoscopy, The Affiliated Tumor Hospital of Guangxi Medical University, Nanning, 530021 Guangxi China

## Correction to: Cell Mol Biol Lett (2021) 26:9 https://doi.org/10.1186/s11658-021-00251-2

Following publication of the original article [1], we have been informed that the authors Hao Chen and Lining Wei were incorrectly affiliated.

The author group has been updated above and the original article [1] has been corrected.
